# Conducting Polymer-Based Cantilever Sensors for Detection Humidity

**DOI:** 10.1155/2018/4782685

**Published:** 2018-06-05

**Authors:** Clarice Steffens, Alexandra Nava Brezolin, Juliana Steffens

**Affiliations:** Department of Food Engineering, URI, Campus of Erechim, Av. Sete de Setembro 1621, 99709-910 Erechim, RS, Brazil

## Abstract

This paper describes the use of different conducting polymers (polyaniline, poly(*o*-ethoxyaniline), and polypyrrole) as a sensitive layer on a silicon cantilever sensor. The mechanical response (deflection) of the bimaterial (the coated cantilever) was investigated under the influence of relative humidity. The variations in the deflection of the coated cantilevers when exposed to relative humidity were evaluated. The results indicated a linear sensitivity in ranges, where the high value was obtained for a polypyrrole-sensitive layer between 20 and 45% of humidity. Furthermore, the sensor shows excellent performance along with rapid response and recovery times, relatively low hysteresis, and excellent stability. The sensors developed are potentially excellent materials for sensing low humidity for long time.

## 1. Introduction

Highly sensitive sensors capable of rapid, real-time, in situ biological and chemical detection are desired. Thus, the sensors based on cantilevers present as miniaturized devices, extremely sensitive to several environmental factors [1], humidity detection [2], biotechnology [3], and agrofood field; the quality control of production and supply chain are currently based on in-depth tests performed in centralized laboratories on a small sample of products.

In this context, there has been a considerable interest in exploring the conducting polymer as a sensitive layer in humidity sensors. Conductive polymers are organic materials having such a structural feature of the combination of monomeric units and can undergo reduction and oxidation reactions. Also, characterized by having electrical properties and magnetic and optical typical metallic behavior but with mechanical properties and processability solution commonly associated with conventional polymers [4, 5].

Conducting polymers suffer many changes when exposed to an analyte, such as modifications of the backbone conformation, solvation effects on the polymer chain, and the attraction of dopant counter ions or transfer of electrons [6]. Thus, changing the electron mobility of charge carriers and on swelling of the polymer matrix is converted into electrical and/or mechanical signals [7, 8]. Among the polymers investigated include the polypyrroles [9, 10], poly(*o*-ethoxyaniline) [11–13], polyaniline [14], and polythiophenes, which offer the greatest possible applications due to their chemical and thermal stability under ambient conditions, processability, ease of synthesis, and doping [15].

Lahav et al. [16] used an electrochemical method to deposit polyaniline on the cantilever surface and detected a deflection on electrochemical oxidation/reduction of the polyaniline film. They observed a mechanical deflection of the cantilever during the redox reactions occurring when exposed to various stimuli.

Steffens et al. [2] evaluated the deflection of the coated (doped and undoped polyaniline) microcantilever under different humidities and observed that the sensors provided good repeatability after several cycles of exposure to RH. Also, the authors evaluated that the response of the microcantilever sensors at different temperatures showed a faster response time at 10°C. The hysteresis of the microcantilever sensor was lower than 2% at the temperatures of 10°C and 20°C [16, 17].

Steffens et al. [18] evaluate the performance of coated microcantilever sensors with polyaniline utilizing the vapors of various volatile organic compounds (methanol, ethanol, acetone, propanol, dichloroethane, toluene, and benzene) with different polarities. The polyaniline layer in the doped state was deposited onto the microcantilever surface, which had been cleaned via plasma. The coated microcantilever sensors were found to have a detection limit range of 17–42 ppm_v_ and response time less than 2.1 seconds.

In this research was study functionalization of silicon cantilever with different conducting polymer layers, aiming the development of humidity sensors. The polymers studied include polyaniline, poly(*o*-ethoxyaniline), and polypyrrole. The technique employed to deposit the polymeric films on the substrates is the spin-coating technique. The linear sensitivity, response time, detection limit, and reversibility of the cantilever sensors were evaluated.

## 2. Materials and Methods

### 2.1. Materials

**Figure 1:**
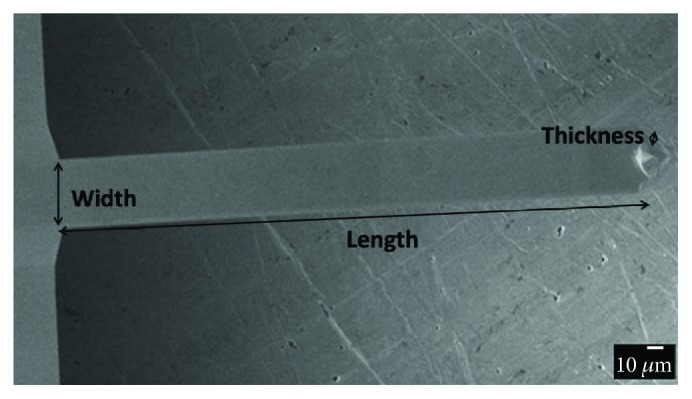
Scanning electron microscopy image of a silicon cantilever. The cantilever has a length of 350 *μ*m, 30.0 *μ*m width, and 0.5–1.5 *μ*m thickness.

The silicon cantilevers were purchased from NT-MDT company, with resonance frequency of 12 (±2) kHz, spring constant of 0.03–0.13 N/m, and dimensions (rectangular shape) of 350.0 *μ*m length; 30.0 *μ*m width, and 0.5–1.5 *μ*m thickness. The cantilever surfaces were cleaned by employing plasma “sputtering” in a high vacuum. The argon gas pressure was lower than 0.1 mbar, and the background pressure was 0.1 mbar. As experimental variables, we employed a radio frequency of 40 kHz, power of 150 W, and treatment temperature of 130°C. Subsequently, the cantilevers were dried in an oven at 50°C for 10 h and stored in a vacuum desiccator. A scanning electron microscopy image of a cantilever used is shown in Figure 1.

The poly(*o*-ethoxyaniline) was obtained by chemical synthesis as described by Mattoso et al. [19]. The chemical synthesis was performed by dissolving the monomer in hydrochloric acid (HCl, 1 M), cooled at 0°C, and mixed to obtain a homogeneous solution. The ammonium persulfate was also dissolved in 1 M HCl, cooled to 0°C, and added to the solution containing the monomer. After reaction for 2 h, the precipitate product was filtered under vacuum and washed with acetone and 1 M HCl in abundance. Afterwards, the polymer was dried under vacuum for 24 h.

The polyaniline was obtained in the emeraldine base oxidation state by an interfacial synthesis followed by a chemical route to obtain the polyaniline nanofibers as reported by Huang and Kaner [20]. The aniline (monomer) was dissolved in an organic solvent (dichloroethane) with the oxidant agent (ammonium persulfate) in 1 M HCl. Then, the oxidant solution was slowly added to the monomer solution in order to avoid mixing of the phases. After 2 h of reaction, the solution was filtered (filter paper Millipore 25 *μ*m) and washed with methanol and Milli-Q water.

Commercial polypyrrole (Aldrich) was used, and its pH was adjusted to 2 with 1 M HCl. All reagents used had analytical grade P.A. and were used without any further purification.

In order to obtain the solution of polyaniline and poly(*o*-ethoxyaniline), the polymers synthetized were dissolved in 5 mL of N-methyl pyrrolidone; these solutions are kept in an ultrasound (Branson) for 30 min for solubilization.

### 2.2. Coating Cantilever with Different Polymers

The AFM cantilever was functionalized with different polymers (polyaniline, poly(*o*-ethoxyaniline), and polypyrrole), all doped with HCl, and thin filmed by the *spin-coating* method, as reported previously by Steffens et al. [2]. All cantilever sensors were dried in a desiccator under vacuum for 12 h at room temperature and finally doped with HCl (1 M).

### 2.3. Cantilever Sensor Performance Measurements

The performance of the cantilever sensor was evaluated by measuring the deflection of the coated in an AFM. The cantilever sensor deflections were measured in voltage, with resolution of millivolts (mV), through monitoring the position of the laser beam, which is focused at the endpoint of the cantilever and reflected to a four-quadrant photodiode, and this signal value was converted in nanometers. All measurements of deflection were performed in triplicate at a static mode of operation.

The cantilever sensor deflection was measured at various relative humidities (RH) in a closed chamber (9 mL) in the static mode. The humidity in the chamber was obtained by combining flows of dry nitrogen (RH from 70 to 20%) and wet nitrogen passing by gas bubbler tube containing water (RH from 20 to 70%) at a flow rate of 0.1 L/min (analog flow mass controller). The temperature in the chamber during the experiments was maintained constant at 20 ± 0.2°C using an ultrathermostatic bath (Nova Ética, 521/2D model).

The reversibility (*η*) of the cantilever sensors at different humidities was calculated as follows [21, 22]:

(1) $$\begin{array}{c} \eta =\frac{D-D_{f}}{D-D_{0}}*100, \end{array}$$

where *D*_0_ is the initial deflection of the microcantilever sensor obtained at 20% of humidity, *D* is the deflection after exposure to a flow of dry nitrogen, and *D*_f_ is the minimum deflection after exposure to wet nitrogen using a water bubbler. The initial deflection of the cantilever sensors was 10 nm during the three cycles evaluated. The reversibility was calculated to assess whether the ability of the sensors varies its state of deflection under the action of humidity and returns to its initial state when the humidity left to act on the sensitive coating. The linear sensitivity of the cantilever sensor was calculated from the slope of the deflection (nm) versus humidity (20–70%) curve.

The sensor hysteresis was calculated as the difference between the mean measured values during the drying and wetting stages.

Detection limits was calculated as the lowest concentration of humidity giving a signal of three times the medium-term deflection stability of the sensor according to the International Union of Pure and Applied Chemistry (IUPAC).

### 2.4. Statistical Analysis

The reversibility and detection limit of the cantilever sensors were expressed as means ± standard deviation and subjected to one-way analysis of variance (Tukey) test at 5% significance using Statistic 5.0 (StatSoft Inc.®, USA) software.

## 3. Results and Discussion

**Figure 2:**
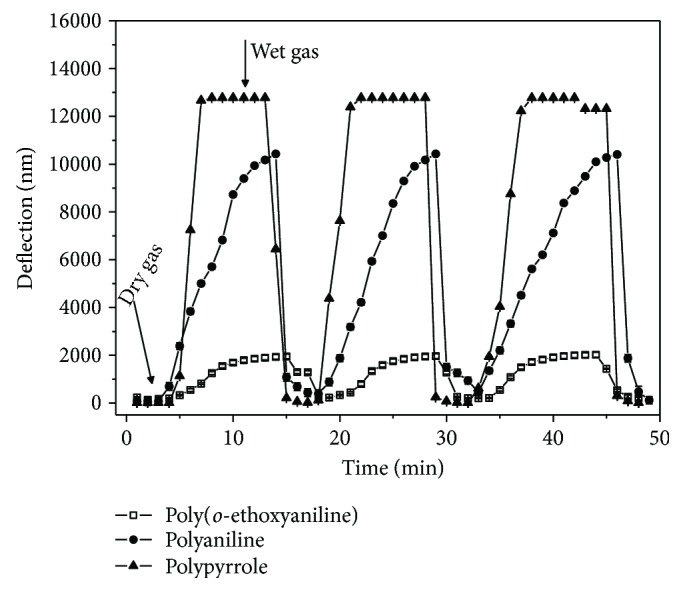
Deflection of cantilever sensors coated with different sensitive layers of conducting polymers (polyaniline, poly(*o*-ethoxyaniline), and polypyrrole) in response to humidity.

The deflection response (nm) of the cantilever sensors was evaluated varying the humidity over a large range (RH = 50%, from 20% to 70%) during desiccation and humidification cycles (wet and dried gas).  Figure 2 shows the cantilever sensor response to humidity, starting from an initial baseline deflection of approximately 0.2 nm. After exposure to dry gas, the deflection of the polypyrrole sensor dramatically increased, in comparison with the polyaniline and poly(*o*-ethoxyaniline), then decreased upon return to wet gas. The sensor responses to humidity were completely reversible to all cycles evaluated (Table 1), also experimentally, were verified when the cantilever sensor coated with polypyrrole exposed to dry gas after a few seconds reached the maximum reading of the photodetector (12 V). Thus, the authors state that it is not a saturation of the sensor response but rather of the AFM photodetector, when it reached 20% RH.

In Figure 2, with increase of humidity, the cantilever deflections up to photodetector voltage and the opposite effect were noted using wet conditions. Thus, the cantilever bending can be correlated with the surface stress changes. Conducting polymers are very sensitive to water vapor and present swelling effects that can be associated with the tensile and compressive interfacial stress change of the adsorption/desorption molecules, respectively.

The detection limit of sensors with different conducting polymers is as low as 170 ppb_v_. The results demonstrated that the cantilever sensor functionalized with polypyrrole presented the higher reversibility and detection limit to humidity (Table 1), differ significantly (*p* < 0.05) to other sensors. The polypyrrole film conduction is of p-type, thus, molecules of water act as electron acceptors attracting the polymer electrons, increasing the conductivity [23]. Also, studies demonstrate that at low humidity the polymer chains would tend to curl up into compact coil form and at high humidity the polymer chains get hydrated by the water absorption [24].

Yang et al. [25] evaluated capacitive microhumidity sensor integrated with a five-stage ring oscillator circuit on chip using the complementary metal oxide semiconductor (CMOS) process. The film of the sensor is polypyrrole, which is prepared by the chemical polymerization method, and the film has a porous structure. Tabard-Cossa et al. [26] evaluated a microactuator device by electrodepositing a polypyrrole film onto the gold-coated side of an AFM microcantilever. It was observed that the volume change of the polypyrrole film is responsible for the mechanical motion [27]. By comparing the results with the present study, a tensile and compressive interfacial stress change on functionalized cantilever exposed to different values of RH was also observed.

Steffens et al. [2] observed when having a desorption of water vapor from the polymer film, a repulsion and swelling of polymer chains occurred, causing a tensile stress. On the other hand, the absorption shrinkage in the polymer film results in a compressive stress.

**Figure 3:**
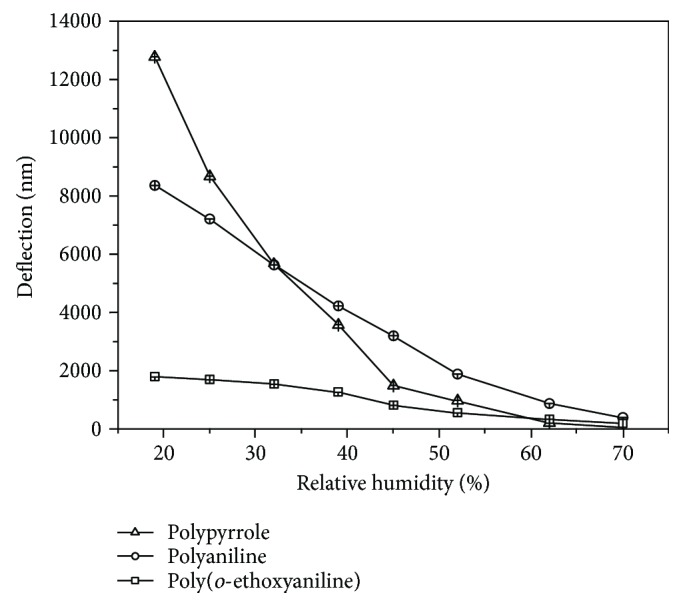
Cantilever deflection with conducting polymers (polyaniline, poly(*o*-ethoxyaniline), and polypyrrole) as a function of relative humidity.

The results obtained in the concentration range from 20 to 70% of RH of cantilever sensors with different conducting polymers were shown in Figure 3. To evaluate the sensitivity of the sensor with different polymers, we calculate the slopes in different ranges, in the piecewise linear approximation, according to Skoog et al. [28]. In observing the slope between 20 and 45% of humidity, the polypyrrole showed the high sensitivity in relation to the other polymers. However, between the ranges of 45 to 70% the sensitivity decreased for all sensors evaluated (Table 2). These differences in the sensitivity can be related to chemical interactions, such as molecular recognition and adsorption/absorption processes of the analyte in the polymer, which cause physical changes, such as swelling and conformational changes in the polymer chains.

**Figure 4:**
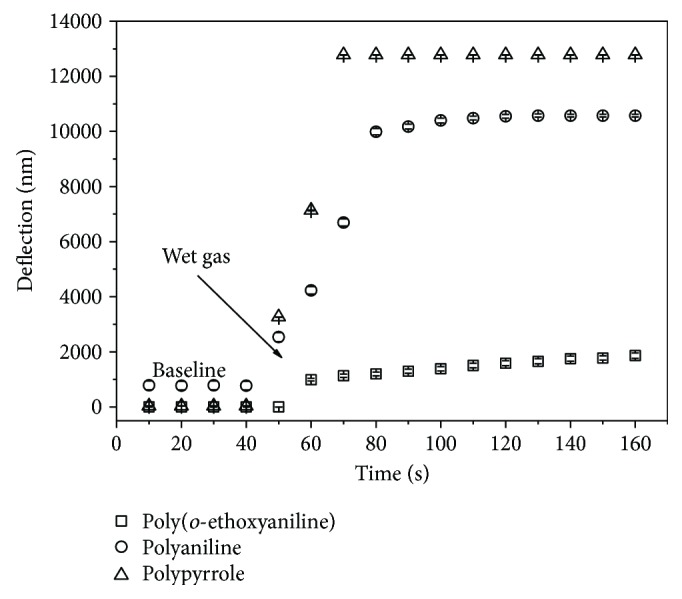
Response time of the cantilever sensors coated with different conducting polymers (polyaniline, poly(*o*-ethoxyaniline), and polypyrrole) to the humidity.

The response time of the cantilever sensors coated with different conducting polymers was obtained when the sensor has reached a steady state from the time where the RH changed. The experiments were carried out to obtain a baseline and sequentially the insertion of wet gas in the chamber; the RH ranges from 20 to 50% at 20°C (Figure 4). The cantilever sensors coated with polyaniline, poly(*o*-ethoxyaniline), and polypyrrole displayed a response time of 5, 10, and 4 s, respectively. It can be seen that the sensors functionalized with poly(*o*-ethoxyaniline) showed the slower response to RH. This is an indication that this polymer showed a great adsorption and desorption of water vapor molecules. Moreover, this shows a fast response time compared with resistive sensors which mostly range from 10 to 30 s at RH [29]. The response time is very high in that of conventional humidity sensor based on ordered macroporous silicon [30], capacitive humidity sensors based on silicon nanowires [31], and low humidity based on the quaternized polypyrrole composite film [32].

Geng et al. [33] evaluated the effect of polymerization time on the humidity sensing properties of polypyrrole and observed a response time of 80 s by increasing from 11 to 95% RH. Compared to the results obtained in this work, one can verify that the cantilever sensor functionalized polypyrrole showed a shorter response time.

**Figure 5:**
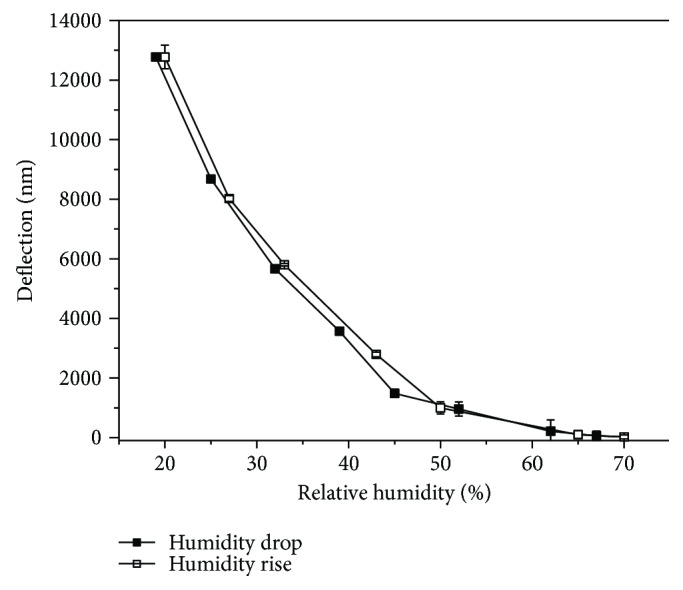
Humidity hysteresis curves for the cantilever sensors coated with polypyrrole.

The humidity hysteresis was analyzed only for the cantilever sensor functionalized with polypyrrole. The humidity was decreased from 70 to 20% (by introducing dry nitrogen into a chamber) and increased from 20 to 70% (wetting nitrogen by passing through water at the same rate of 0.1 L/min) (Figure 5). The hysteresis was calculated as the difference between the mean measured values during the drying and wetting stages. The deflection of the cantilever humidity sensor during 8 consecutive cycles of wetting and drying showed 1.23% hysteresis.

Thus, it is possible to observe that conducting polymers used (polypyrrole, polyaniline, and poly(*o*-ethoxyaniline)) present many advantages in comparison to metal oxide and other humidity sensors due to their capacity to operate at room temperature at low cost.

**Figure 6:**
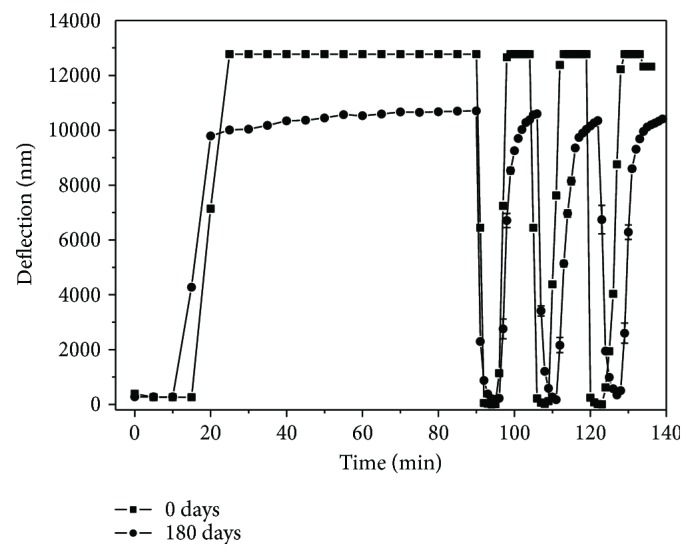
Response of the cantilever sensors coated with polypyrrole after 0 and 180 days.

The cantilever sensor coated with polypyrrole film who presented better response to humidity was stored for 180 days and evaluated the response at RH (Figure 6). The results demonstrate that the response remained with time (after 180 days) although with sensitivity decrease of 160% in relation to the initial. Thus, the sensitive layer of polypyrrole presented a good stability at environment conditions.

## 4. Conclusions

Different conducting polymers were applied to develop a humidity sensor using a microeletromechanical system (cantilever). The polypyrrole cantilever sensor presented very low detection limit and good reproducibility compared to polyaniline and poly(*o*-ethoxyaniline) sensors to RH. The hysteresis of the sensor was found as 1.23% RH, and the response time measurements showed 3 seconds. The sensors developed are potentially excellent materials for sensing low humidity for long time.

## Figures and Tables

**Table 1:** Values of reversibility (%) and detection limit (LOD) of the cantilever sensor coated with different conducting polymers.

Sensitive layer	Reversibility^∗^ (%)	LOD^∗^ (ppb_v_)
Polypyrrole	99.83^a^ ± 0.01	17.00^c^ ± 4.00
Polyaniline	99.80^b^ ± 0.01	100.02^b^ ± 25.00
Poly(*o*-ethoxyaniline)	99.02^c^ ± 0.04	170.01^a^ ± 23.00

^∗^Means followed by the same letters on a column represent no significant difference at 5% level (Tukey's test).

**Table 2:** Sensitivity in the piecewise linear approximation, for the humidity cantilever sensor coated with different conducting polymers.

Sensitive layer	*R* ^2^	Slope (nm/% RH)	Linear range of % RH
Polypyrrole	1	682.6	20–25
	1	430.7	25–30
	0.99	320.8	30–45
	1	70.1	45–62
	1	30.2	62–70
Polyaniline	1	197.8	20–52
	1	100	52–62
	1	61.2	62–70
Poly(*o*-ethoxyaniline)	0.99	26.4	20–40
	1	74.2	40–45
	0.99	24.1	45–70

## Data Availability

The data used to support the findings of this study are available from the corresponding author upon request.

## Supplementary Materials

Supplementary Materials Graphical abstract: the figure shows the cantilever behavior (deflection—nm) when submitted at different humidities using dry and wet nitrogen gas. The high sensitivity can be due to the tensile and compressive interfacial stress change of the adsorption/desorption molecules by the conducting polymer deposited over the cantilever surface.
