# Supplementary material for: Conducting Polymer-Based Cantilever Sensors for Detection Humidity
Source: Scanning. 2018 Jun 5;2018:4782685. doi: 10.1155/2018/4782685 (PMC6008807; doi:10.1155/2018/4782685)

Graphical Abstract

The Figure 1 shows the cantilever behavior (deflection - nm) when submitted at different humidity's using dry and wet nitrogen gas. The high sensitivity can be due the tensile and compressive interfacial stress change of the adsorption/desorption molecules by the conducting polymer deposited over the cantilever surface.

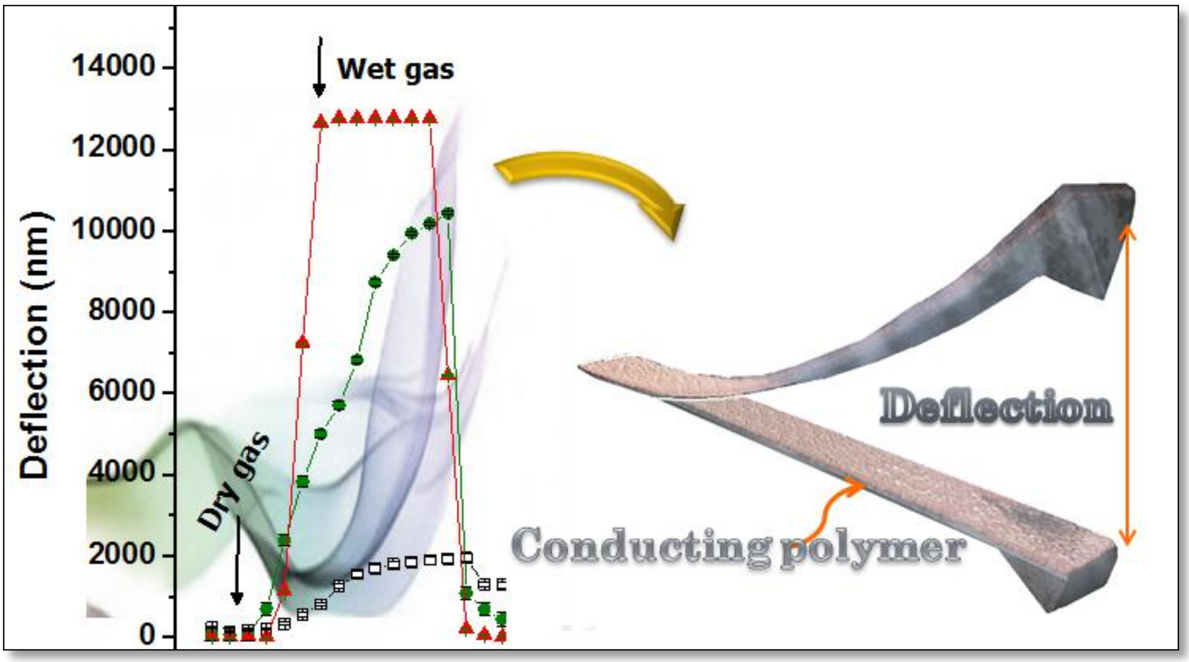

Supplement: Supplementary Materials — Graphical abstract: the figure shows the cantilever behavior (deflection—nm) when submitted at different humidities using dry and wet nitrogen gas. The high sensitivity can be due to the tensile and compressive interfacial stress change of the adsorption/desorption molecules by the conducting polymer deposited over the cantilever surface. [file 4782685.f1.pdf]
